# Present and future suitability of invasive and urban vectors through an environmentally driven mosquito reproduction number

**DOI:** 10.1098/rspb.2024.1960

**Published:** 2024-11-06

**Authors:** Marta Pardo-Araujo, Roger Eritja, David Alonso, Frederic Bartumeus

**Affiliations:** ^1^ Centre d’Estudis Avançats de Blanes (CEAB-CSIC), Blanes, Spain; ^2^ Institució Catalana de Recerca i Estudis Avançats (ICREA), Barcelona, Spain; ^3^ Centre de Recerca Ecològica i Aplicacions Forestals (CREAF), Barcelona, Spain

**Keywords:** *Aedes*, climate change, invasive species, mosquito population dynamics, vector-borne diseases, vector ecology

## Abstract

Temperature and water availability significantly influence mosquito population dynamics. We have developed a method, integrating experimental data with insights from mosquito and thermal biology, to calculate the basic reproduction number ($R_{M}$) for urban mosquito species *Aedes albopictus* and *Aedes aegypti*. $R_{M}$ represents the number of female mosquitoes produced by one female during her lifespan, indicating suitability for growth. Environmental conditions, including temperature, rainfall and human density, influence $R_{M}$ by altering key mosquito life cycle traits. Validation using data from Spain and Europe confirms the approach’s reliability. Our analysis suggests that temperature increases may not uniformly benefit *Ae. albopictus* proliferation but could boost *Ae. aegypti* expansion. We suggest using vector $R_{M}$ maps, leveraging climate and environmental data, to predict areas susceptible to invasive mosquito population growth. These maps aid resource allocation for intervention strategies, supporting effective vector surveillance and management efforts.

## 1. Introduction

Insects exhibit complex life cycles and respond rapidly to environmental changes, making it challenging to track their population dynamics. Species like dragonflies, mosquitoes and moths, for example, undergo life cycles that span aquatic, terrestrial and aerial stages. Their rapid dynamics are influenced by factors such as weather conditions, water availability, landscape features and human activities [1]. Consequently, insect population dynamics tend to be bursty and difficult to trace, displaying highly heterogeneous patterns across both space and time.

Among extensively studied insect species, mosquitoes stand out due to their capacity to transmit global diseases such as malaria and dengue. Modelling insect dynamics, particularly mosquitoes, demands a substantial volume of data. Commonly employed data types include presence–absence records and insect trap counts, both incurring significant costs and often inadequately covering the entire distribution of the species. Given these limitations, mathematical approaches become paramount for extrapolating the spatial and temporal evolution of a species.

Numerous mathematical frameworks have been employed to model insect dynamics [2]. One notable approach, mostly used for mosquitoes, relies on the principles of thermal biology [3]. This approach explores the relationships between temperature and key traits governing the mosquito’s life cycle [4–7]. The thermal biology approach can be integrated with the concept of the basic reproduction number, $R_{0}$ [8], to reveal how mosquito numbers vary in response to temperature fluctuations. In the context of diseases, the basic reproduction number, $R_{0}$, is defined as the number of secondary infections in an otherwise susceptible population caused by a single original infection. For the case of mosquitoes [9], the basic reproductive number is defined as the number of female mosquitoes produced by a single female mosquito in its entire lifespan, $R_{M}$. The application of these frameworks to vector population dynamics has remained largely unexplored.

Empirical evidence underscores the pivotal role of temperature in the mosquito life cycle [10,11], temporal dynamics [12,13] and spatial distribution [14]. Beyond temperature, mosquito population studies reveal the influence of factors such as rainfall [15,16], human population density [17], land cover [18] and relative humidity [19,20]. In this study, we investigate how temperature, rainfall and human population density modulate $R_{M}$, serving as an indicator of mosquito suitability for growth. If $R_{M}>1$, the female mosquito population experiences exponential growth over time, resulting in an overall population increase. If $R_{M}<1$, the female mosquito population steadily diminishes until it reaches extinction. In other words, $R_{M}$ indicates whether mosquito proliferation is likely to occur after colonizing a specific area, based on the environmental conditions present in that location. We concentrate on two species of *Aedes* mosquitoes: *Aedes albopictus* and *Aedes aegypti*, potential vectors for diseases including dengue, Zika, chikungunya or yellow fever. *Ae. albopictus* has established populations across Europe, from east to west. It has been present in Albania since 1979 [21] and in Spain since 2004 [22]. *Ae. aegypti* is not currently established in Europe, except on Madeira Island (Portugal), but is present in the eastern Black Sea region [23]. Nonetheless, it sporadically arrived in the Canary Islands, primarily from Madeira and Cape Verde Islands (Africa), with documented records of adult and larval presence triggering local surveillance and control campaigns in Spain [24]. Additionally, *Ae. aegypti* has been observed in the Netherlands [25], Cyprus [26] and on the Crimean peninsula in Ukraine [23]. To validate our approach, we calculate the environmentally driven mosquito reproduction number for *Ae. albopictus* and compare it with presence–absence and trap count data in Spain, as well as with presence–absence data across Europe. We also illustrate the differing spatial distributions of the invasive species *Ae. albopictus* and *Ae. aegypti*, and evaluate the impact of future climate change scenarios on the mosquito reproduction number.

As emphasized by the World Health Organization, the absence of established vaccines underscores the critical role of vector management in addressing the growing global burden of vector-borne diseases [27]. This issue is further exacerbated by global changes that facilitate the establishment of new invasive vector species, with mosquitoes being particularly sensitive to shifts in climate and land use [28,29]. In this context, introducing an innovative suitability index to cast hotspots of vector suitability for growth (and thus establishment) can facilitate the implementation of proactive and cost-efficient vector management measures.

## 2. Material and methods

### (a) Data

To calculate the mosquito basic reproduction number, $R_{M}$, we modelled the parameters of the mosquito life cycle as a function of three environmental variables: temperature, rainfall and density of the human population. We then validate the mosquito basic reproduction number as a suitability index using occurrence and trap count data.

#### (i) Climatic data

Historical climatic data for 2004 and 2020, including temperature and rainfall, were extracted from the CERRA dataset (ECMWF Re-Analysis 5 [30]). CERRA ERA5 is a freely accessible dataset offering climatic data from 1940 until July 2021, with a temporal resolution of 3 h and a spatial resolution of 5.5 km.

Monthly climate data for future predictions for the periods 2041–2060 and 2061–2080 were extracted from the CMIP6 dataset with a resolution of 2.5 min of a degree of longitude and latitude (source: https://worldclim.org/data/cmip6/cmip6climate.html). We used the R package *geodata* [31] to extract the data. We chose the SSP3-7.0 socioeconomic pathway, which defines the climate change scenario ‘Middle of the Road’ which represents an intermediate mitigation effort against climate change. We extracted the two variables related to temperature available on the WorldClim website: monthly average minimum and maximum temperature. We then computed mean temperature as the average of these two variables. We use this average as an approximation for the mean temperatures, acknowledging that, while it represents the best available estimate, it is not an exact measure. WorldClim offers multiple datasets derived from CMIP6. To mitigate biases originating from specific datasets available in WordClim, we download all of them and compute the mean across all datasets. These averaged values were then used as representative values for each period and location.

#### (ii) Human density data

We obtained the Spanish human population density data from the INE (Instituto Nacional de Estadística) at yearly and municipality resolution. We extracted European human population data from the fourth version Gridded Population of the World (GPW) collection at 2.5 arc-minute (aproximately 5 km) for the year 2020 [32]. For future scenarios, we used population density estimates from the GHS datasets [33] for 2030 (source: https://ghsl.jrc.ec.europa.eu/ghs_pop2023.php).

#### (iii) Entomological data

We validated the $R_{M}$ computation for *Ae. albopictus* using multiple occurrence datasets, one for Europe and one for Spain with finer resolution. The data sourced from citizen science and official data sources.

Occurrence data spans the entire invasion process of *Ae. albopictus* in Spain from 2004 up to October 2023 [34]. These data are sourced from official data sources, which involved counts of physical specimens collected by specialized organizations, at the municipal level, mostly conducted through oviposition and adult traps. The adult traps are BG Sentinel2 (BioGents; https://eu.biogents.com/), while the oviposition traps are 300 ml black plastic cups with a substrate of Mansonite or tongue depressors.

As an extended exercise, we also validated the $R_{M}$ for *Ae. albopictus* in Spain with trap count data [34]. The Spanish mosquito trap count data originated from a total of 13 BG Sentinel2 traps distributed across various cities in the northeastern Spain (Catalonia region) with the following number of traps per location: Blanes (11), Lloret de Mar (1), Palafolls (1), Tordera (1). The traps operated from November 2020 until December 2022 for Blanes, from September 2020 until December 2022 for Palafolls, from May 2020 until December 2022 for Todera, from June 2020 until November 2020 for Lloret de Mar, and mosquito samples were collected every week.

#### (iv) Citizen science data

The occurrence data for the invasion process of *Ae. albopictus* in Spain from 2014 up to October 2023 also combine citizen observations [34] coming from the Mosquito Alert platform [35,36] (see daily updated maps in Europe at https://map.mosquitoalert.com/spa/distribution/en). Specifically, the validation involved *Ae. albopictus* occurrence data at the municipal level in five administrative regions of Spain (Andalusia, Aragon, Catalonia, Valencian Community, Basque Country). At the European scale, we validated the $R_{M}$ computation using occurrence data from October 2023 combining two different sources: the ECDC agency and the Mosquito Alert citizen science platform. The data at the European scale corresponds to administrative units at the NUTS 3 level, roughly matching the province level, hence coarser resolution than for the Spanish analysis.

### (b) Vector dynamic model

We have constructed a deterministic compartmental model to capture the dynamics of the mosquito life cycle [13,37,38]. The model incorporates three distinct stages: egg (E), immature (I) and adult mosquito (A). The larva and pupa stages are combined into a single immature compartment for simplicity. Model parameters, including development, mortality and fecundity rates, depend on temperature [10,39], while the hatching rate and carrying capacity of the immature stage are influenced by rainfall and human density. The latter parameters are particularly relevant to the aquatic phases of mosquito development. Rainfall directly affects the availability of natural breeding sites, and human density is expected to positively correlate with the proliferation and maintenance of water containers resulting from human activities, serving as mosquito breeding sites [40,41].

The temporal evolution of each variable is governed by the following equations


(2.1) $$\begin{array}{rl} \frac{dE}{dt} & =afA-d_{E}hE-\delta_{E}E\\ \frac{dI}{dt} & =d_{E}hE-d_{I}I-\delta_{I}(1+\frac{I}{K})I\\ \frac{dA}{dt} & =d_{I}I-\delta_{A}A, \end{array}$$

where $\delta_{X}$ is the mortality rate and $d_{X}$ is the development rate for the X compartment. The number of offspring laid by a female mosquito per gonotrophic cycle is denoted by f, while a defines the inverse of the length of the gonotrophic cycle, which is a surrogate of the biting rate. The proportion of eggs hatching is given by h and we define larval resource limitations in the form of a carrying capacity K. The underlying assumption is that larvae compete for water availability and resources, being these limiting factors that determine the number of surviving larvae that can develop to the adult stage.

All model parameters depend on temperature, rainfall and human density (see table 1). Parameters fall into two groups: one influenced exclusively by temperature, based on species-specific laboratory data and showing interspecies variations [5], and a second group that includes hatching rate, a parameter that is consistent across species and depends on human density and rainfall. We computed the parameters that depend on temperature using methods akin to prior work [5,42], combining thermal biology response functions with laboratory data [10,11,39,43–47]. For some parameters, we obtained the functional form from Mordecai *et al*. [5], while we computed the functional form for other parameters that were not available: egg development rates, egg mortality rates and the probability from larva to adult stage for both species (see electronic supplementary material, section 4, for more details).

**Table 1.** Mosquito model parameters as a function of temperature (T), rainfall (R) and human density (H). See electronic supplementary material, table S2, for the specific functional forms of the parameters.

parameter	definition
$a^{-1}=a^{-1}\,\left(T\right)$	average length of the gonotrophic cycle
f=f(T)	fecundity rate (no. of eggs per gonotrophic cycle)
K=K⁢(R,H)	carrying capacity
h=h⁢(R,H)	proportion of eggs that successfully hatch
$d_{I}=d_{I}\,\left(T\right)$	development rate from larva to adult mosquito
$d_{E}=d_{E}\,\left(T\right)$	development rate from egg to larva
$\delta_{E}=\delta_{E}\,\left(T\right)$	egg mortality rate
$\delta_{I}=\delta_{I}\,\left(T\right)$	immature mortality rate
$\delta_{A}=\delta_{A}\,\left(T\right)$	adult mortality rate

We assume that the functional form for the hatching rate does not differ between *Ae. aegypti* and *Ae. albopictus*. The hatching rate, which describes the proportion of hatching eggs as a function of water container availability, dependent on human density and rainfall, is sourced from Metelmann *et al.* [48] and is given by


(2.2) $$h(R,H)=(1-\epsilon_{rat})\frac{\left(1+\epsilon_{0})\;\exp\;(-\epsilon_{var}(R(t)-\epsilon_{opt}{)}^{2}\right)}{\exp((-\epsilon_{var}(R(t)-\epsilon_{opt}{)}^{2})+\epsilon_{0}}+\epsilon_{rat}\frac{\epsilon_{dens}}{\epsilon_{dens}+\exp(-\epsilon_{fac}H(t))}$$

where H is the human density in square kilometres and R is the rainfall in mm. The hatching rate surpasses 0.99 for values of rainfall between 7.2 and 8.7 mm and human density above 850 people per square kilometre, electronic supplementary material, figure S3. Equation (2.2) defines the proportion of eggs that hatch as a function of rainfall and human density. The first summand represents rainfall impact, following a unimodal pattern; both low and excessive rainfall hinder hatching. The second summand reflects human density influence, saturating at optimal levels (electronic supplementary material, figure S3). The coefficient $e_{rat}$ (set at 0.5 for equal weighting) regulates variable importance, specifically human density and rainfall, in hatching rate.

### (c) Mosquito basic reproduction number

The mosquito basic reproduction number, $R_{M}$, defines the average number of adult female mosquitoes produced by one female mosquito in her entire lifespan [9]. Consequently, the value of $R_{M}$ determines whether the population of female adult mosquitoes will increase or decrease in a given region, conditioned to the existence of an incipient colonizing population.

If $R_{M}>1$, the population increases and approaches its maximum capacity, limited by the carrying capacity K. On the contrary, if $R_{M}<1$, the population goes extinct, resulting in the absence of adult mosquitoes.

To compute this number, we utilized the method proposed by Diekmann *et al.* [8], defining the basic reproduction number as the leading eigenvalue of the next generation matrix (*NGM*). From the Jacobian matrix of the system (see electronic supplementary material, section 1, for more details), we can compute $NGM=-T\Sigma^{-1}$,


(2.3) $$\begin{array}{rl} NGM & =\left(\begin{array}{ccc} 0 & 0 & af/(d_{E}h+\delta_{E})\\ d_{E}h/(d_{I}+\delta_{I}) & 0 & 0\\ 0 & d_{I}/\delta_{A} & 0 \end{array}\right) \end{array}$$

where the leading eigenvalue of this matrix determines the mosquito basic reproduction number,


(2.4) $$R_{M}=\sqrt[{3}]{f\frac{a}{\delta_{A}}\frac{d_{E}h}{\left(d_{E}h+\delta_{E}\right)}\frac{d_{I}}{\left(d_{I}+\delta_{I}\right)}}=\sqrt[{3}]{f\frac{a}{\delta_{A}}p_{EL}p_{LA}}.$$

where $p_{EL}$ defines the probability to develop from egg to larvae and $p_{LA}$ from larvae to adult. The $R_{M}$ is the geometric mean of the transition probabilities of each of the populations in the model, with the fecundity rate as an additional factor.

It is important to note that the carrying capacity does not appear in the calculation of $R_{M}$, which is why we do not explicitly define this parameter. Additionally, the calculation of $R_{M}$ does not account for transportation factors and assumes that an initial arrival has already occurred in an area with no existing mosquitoes. Therefore, we should consider $R_{M}$ as a vector suitability index rather than a true invasibility index.

### (d) Validation

To validate the suitability index, $R_{M}$, we used occurrence data for *Ae. albopictus* in Spain (see figure 3*c*) and Europe (see electronic supplementary material, section 8, figure S9). Additionally, we employed count data for *Ae. albopictus* in several cities of Spain, located near the coast at the border between Barcelona and Girona provinces (see §2a for more details).

The occurrence map of *Ae. albopictus*, figure 3*a*, in Spain should be understood as a representation of vector establishment as of the year 2023, at the municipality level. This map indicates the initial detections of the invasive mosquito in various municipalities as it spread across the territory over time. It is important to note that these initial detections should not be interpreted as actual colonization events. Colonization events, which are often linked more to transportation dynamics than climatic factors, are frequently overlooked. Instead, these initial detections are more indicative of the early or potentially delayed establishment of mosquito populations. This establishment process is closely associated with thermo-biological responses, as reflected in the $R_{M}$ values.

For validation purposes, we aggregated the occurrence data by region (NUTS2) and computed the percentage of municipalities (the smallest administrative unit) showing established populations of *Ae. albopictus*. Next, we computed the monthly $R_{M}$ using monthly average temperature and monthly mean daily rainfall, along with the annual average human density for the period 2003–2020. This period included 1 year before the first record of *Ae. albopictus* in Spain (2003) until the last year available in the CERRA ERA5 dataset (2020). Finally, we aggregated municipalities by the number of suitable months ($R_{M}>1$) for *Ae. albopictus* within the season and assessed, for each suitability value (from 1 to 12 months), the percentage of municipalities with established populations of *Ae.albopictus*.

We also compared weekly mosquito trap counts in different locations with locally computed $R_{M}$s for each trap location and week. We compute the $R_{M}$ using the weekly mean temperature and mean daily rainfall at CERRA cells where the traps were located. We used a generalized linear model (GLM) with a Poisson error distribution with explanatory variable $R_{M}$ and response variable the mosquito female counts. We plotted the results along with the 95% confidence interval and display the results in the electronic supplementary material, table S4.

## 3. Results

### (a) Thermo-dependent mosquito basic reproduction number

**Figure 1.**
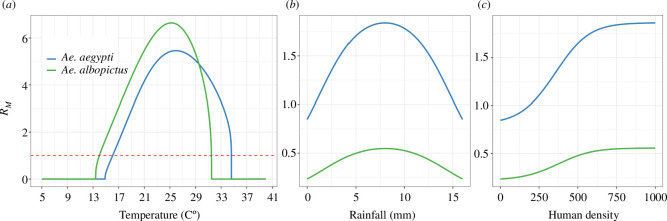
(*a*) The mosquito basic reproduction number, $R_{M}$, for the two species *Ae. albopictus* and *Ae. aegypti* as a function of temperature with constant human density (*H* = 500) and rainfall (*R* = 8 mm). (*b,c*)$R_{M}$ as a function of (*b*) rainfall and (*c*) human density with constant temperature (*T* = 16°C) and (*b*) *H* = 0 or (*c*) *R* = 0, respectively, to show the effect of each variable. We assumed the same functional form for rainfall and human density for the two species.

The mosquito basic reproduction number, $R_{M}$, is species-dependent and varies as environmental variables vary across time and space. Each of the two *Aedes* species showed a distinct thermal optimum and suitable (i.e. $R_{M}>1$) temperature range. The differences among species suggested differential reproductive capacities associated with temperature (figure 1*a*). In the calculation of $R_{M}$, the proportion of hatching eggs depended on water availability. This parameter was determined using equation (2.2) as described by Metelmann *et al*. [48], thus being influenced by precipitation and human density, as illustrated in figure 1*b,c*. We applied the same equation for both species, assuming a similar biological response to precipitation and human density. However, it is worth noting that these environmental variables played a crucial role in defining the spatial variation in the mosquito basic reproduction number.

By isolating the temperature effect we can better comprehend how temperature uniquely influences each species. We fixed the rainfall and human density to its optima (where the hatching rate, expressed as a proportion, is one, R=8mm and H=500). For *Ae. albopictus*, the suitable temperature range (where $R_{M}>1$) is narrower (14.0–31.5°C) in comparison with *Ae. aegypti*, and the optimum temperature for proliferation is reached at 25.0° (figure 1*a*). *Ae. aegypti* suitability range spans over warmer temperatures (16.1–34.6°C) and shows an optimum value at 25.9°. In conclusion, the optimal temperature for maximal growth potential in *Ae. aegypti* is nearly 1°C higher compared with *Ae. albopictus*. We also show variations in the suitable temperature range (but not in the optimum temperature) for different values of rainfall and human density (electronic supplementary material, figure S1). Moreover, we conducted a sensitivity analysis to understand the influence of each temperature dependent parameter on the suitability index (see electronic supplementary material, section 5, for more details).

### (b) The condition $R_{M}>1$ to build vector suitability maps

**Figure 2.**
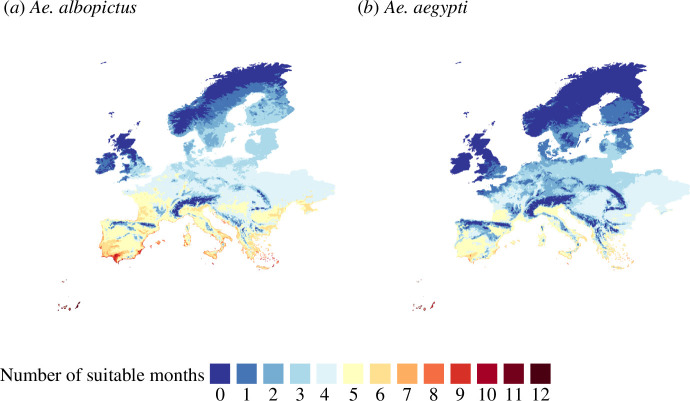
Vector suitability maps (Europe 2020) for (*a*) *Ae. albopictus* and (*b*) *Ae. aegypti*. Suitability is represented as the number of months with $R_{M}>1$. Colour gradient from brown (large number of suitable months) to blue (low number of suitable months).

We can interpret the mosquito basic reproduction number, $R_{M}$, as an indicator of the suitability of mosquito population growth, in this case, modulated by temperature, rainfall and human density. We depicted annual (figure 2) and seasonal (electronic supplementary material, section 6) vector suitability maps for each targeted species for 2020. To build up both annual and seasonal maps, we used monthly averages of temperature and rainfall and average annual human density. Rather than averaging $R_{M}$ over months to obtain an annual picture of vector suitability, we followed Di Pol *et al.* and Mordecai *et al.* [5,6] and computed the number of months when the vector suitability index is greater than one ($R_{M}>1$) within a year (figure 2). The larger the number of months with $R_{M}>1$ in a given region, the easier it is for mosquito populations to establish (compared with another region with the same inflow of mosquitoes).

*Ae. albopictus* showed higher suitability in Europe compared with *Ae. aegypti* (figure 2). *Ae. aegypti* exhibits high suitability in the Mediterranean countries, primarily along the coast, with Spain having the highest suitability for this species. This difference is attributed to the warmer temperature range for *Ae. aegypti* compared with *Ae. albopictus* (figure 1). For the two species, the Mediterranean coast has the highest suitability in almost all of the territory. In addition, the lowest suitability is located in the mountain ranges (e.g. Pyrenees and Alps) and the northeast European countries. We can see that in the Mediterranean areas, there are some regions where *Ae. albopictus* is suitable for nine months. This aligns with recent findings where this mosquito was found in traps during winter months in the east and west Mediterranean countries [49].

### (c) Validation with occurrence and count data of *Ae. albopictus* in Spain

**Figure 3.**
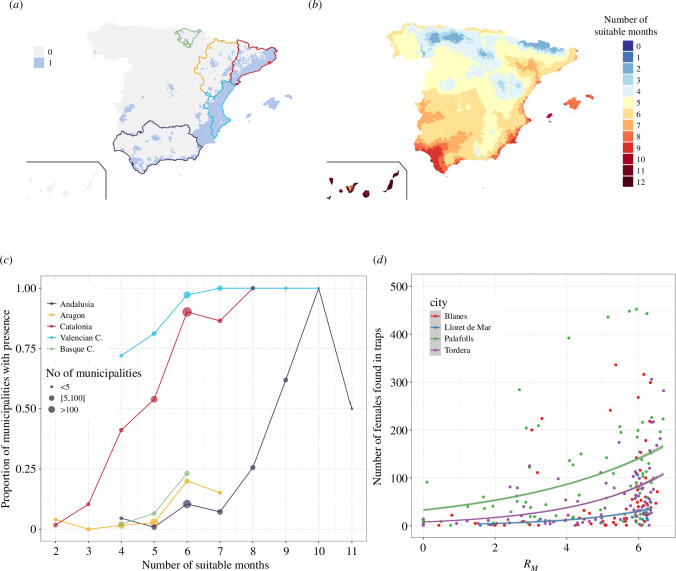
Validation of the mosquito basic reproduction number, $R_{M}$, with empirical data. (*a*) Municipality level presence (blue) and absence (grey) of *Ae. albopictus* in Spain in October 2023. (*b*) Number of suitable months (i.e. $R_{M}>1$) for *Ae. albopictus* in for the period 2003–2020, we compute the $R_{M}$ with the average rainfall, temperature and human density for this period. (*c*) Relationship between $R_{M}$ for six different regions in Spain and the proportion of municipalities that are positive for the presence of *Ae. albopictus* in the regions. Catalonia and the Valencian Community where *Ae. albopictus* has earlier establishment (first records 2004 and 2005, respectively) compared with the initial presence of these mosquitoes in the Basque Country, Andalusia and Aragon (2016, 2014 and 2016, respectively). (*d*) Generalized linear model with response variable the number of female *Ae. albopictus* found in a trap and explanatory variable $R_{M}$ for four different city locations in northeastern Spain (indicated with a star in (*a*)): Blanes, Lloret de Mar, Palafolls and Tordera.

To validate the mosquito basic reproduction number as a vector suitability index, we investigated whether and how the number of suitable months during the period 2003–2020 (i.e. the average $R_{M}$ for most of the spreading period of *Ae. albopictus* in Spain) is associated with the progressive establishment of local populations at the municipality level (figure 3*a–c*). We selected five regions (Comunidades Autónomas) at different invasion stages (figure 3*c*). In Catalonia and Valencia Community, *Ae. albopictus* is fully established in many municipalities with the first detection of the species in 2004 and 2005, respectively [22]. In Andalusia (south), Aragon (northeast, non-coastal) and Basque Country (north) the species was first detected about 10 years later: 2014 (Andalusia) and 2016 (Aragon and Basque Country). As *Ae. aegypti* has not yet colonized Spain, and its current establishment in Europe is limited to a few eastern countries, we did not validate the computation of $R_{M}$ for this species.

In general, the higher the number of suitable months for *Ae. albopictus* the greater the proportion of municipalities with established mosquito populations. In the eastern coastal regions of Spain (Catalonia and Valencian Community), where *Ae. albopictus* first established and spread, over 50% of the municipalities showing an $R_{M}>1$ for at least five months within the season showed established populations of *Ae. albopictus* (figure 3*c*). All municipalities showed full *Ae. albopictus* establishment when $R_{M}>1$ for at least eight months within the season. Conversely, in recently colonized areas like Andalusia, Aragon and the Basque Country, a different pattern emerges as both repeated colonizations and sustained favourable conditions are necessary for full species establishment. Therefore, we only start to see a large proportion of municipalities with established *Ae. albopictus* when $R_{M}>1$ holds beyond seven months during the season. Conversely, suitability indexes below six months were not associated with *Ae. albopictus* establishments. For example, in Andalusia, more than 60% of municipalities showed established populations of *Ae. albopictus* when optimal environmental conditions ($R_{M}>1$) were present for at least nine months of the year.

A similar analysis at the European scale for *Ae. albopictus* (see electronic supplementary material, section 7, figure S9) reveals a correlation between suitable months (i.e. months with $R_{M}>1$) and regions where *Ae. albopictus* populations have been detected and established.

At the local (city) scale, we validated the suitability index, $R_{M}$, with weekly mosquito trap counts (figure 3*d*). In this case, the $R_{M}$ was computed using the mean temperature and mean daily rainfall for each mosquito collection week. Over three seasons (2020–2022) and in four locations (Blanes, Lloret del Mar, Palafolls and Tordera), we observed a significant and positive regression coefficient (figure 3*d*) between the $R_{M}$ for *Ae. albopictus* and weekly mosquito trap counts. For every unit increase in $R_{M}$, we observed a significantly multiplicative effect of (1.41–1.59) on the average number of counts (more details in electronic supplementary material, section 8, table S4).

### (d) Climate change scenarios for *Aedes* invasive species in Spain and Europe

**Figure 4.**
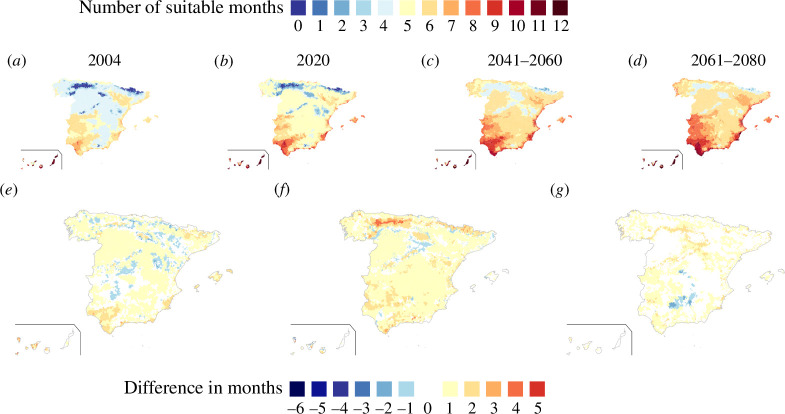
(*a*–*d*) Vector suitability maps of *Ae. albopictus* in Spain for (*a*) 2004, (*b*) 2020, and two climate future projections from CMIP6 dataset for the periods (*c*) 2041–2060 and (*d*) 2061–2080. (*e*–*g*) Maps comparing different suitability maps and showing in colours the difference in suitable months (*e*) between 2004 and 2020, (*f*) between 2020 and 2041–2060, and (*g*) between 2041–2060 and 2061–2080. Blue colours indicate a decrease in suitability, while brown denotes an increase.

**Figure 5.**
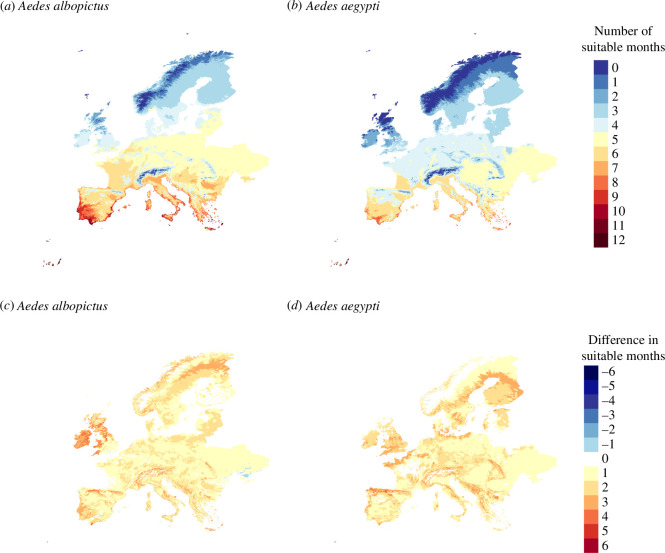
(*a*,*b*) Vector suitability maps for future climate projections in Europe for the period of 2061–2080 for (*a*) *Ae. albopictus* and (*b*) *Ae. aegypti.* (*c*,*d*) Maps showing the difference in suitable months from the period of 2061–2080 and 2020 for (*c*) *Ae. albopictus* and (*d*) *Ae. aegypti*.

We computed the suitability index, $R_{M}$, for future climate projections across Europe for both *Ae. albopictus* and *Ae. aegypti*. It covers the periods 2041–2060 (electronic supplementary material, figure S8) and 2061–2080 (figure 5) For population density, we used estimates for 2030 [33]. We also specifically analysed the suitability index for *Ae. albopictus* in Spain (figure 4). Our comparisons included four different time periods: 2004, marking the first recorded presence of *Ae. albopictus* in northeastern Spain (Catalonia); 2020, the last year covered by the CERRA dataset; and two future climate projections, for the periods 2041–2060 and 2061–2080 (figure 4*c,d*).

The number of suitable months where $R_{M}>1$ in Spain rose from 2004 to 2020 and beyond. *Ae. albopictus* invaded the northeastern Spain (Catalonia) in 2004, coinciding with high suitability in the region (figure 4*a*). In 2020, the number of suitable months also increased in the south of Spain (Andalusia) and in some coastal regions of the northwest (Galicia) (figure 4*e*), coinciding with the more recent invasion process in the south and a first established population in Galicia in 2023. Overall, Spain shows increased suitability for both periods 2041–2060 and 2061–2080, with significant rises in the northern regions, in particular for the 2041–2060 projection (figure 4*f*). Yet, some regions experienced a decline in the number of suitable months from 2004 to 2020 (figure 4*e*) due to scarce rainfall and excessive temperatures. In addition, the central-south regions of Spain experienced a decline in the number of suitable months in the projection 2061–2080, attributed also to excessively high summer temperatures exceeding *Ae. albopictus*’s thermal tolerance (figure 1*a*).

In Europe, *Ae. albopictus* suitability increased, particularly in southern regions, extending the mosquito activity season. Central and northern Europe showed a comparatively smaller rise in suitable months, while Ireland and England showed the most significant increase, with up to four months of suitability. For 2061–2080, the northeast part of Europe showed a projected increase of up to five favourable months for *Ae. albopictus*, in Scandinavia’s southern regions, deviating from the general trend. This climate change scenario may lead to the establishment of *Ae. albopictus* if migration occurs from areas already colonized. Regarding *Ae. aegypti*, we observed an increase in suitability in southern regions of Europe (figure 5*d*), while the northern parts retained fewer suitable months. For certain north-central European regions suitability is bounded to only four months during the period 2061–2080 (figure 5*b*). Northern France showed a more significant increase, with up to a four month rise in suitability, while central France maintained the same level of suitability. Moreover, there was a decrease in suitability resulting from reduced rainfall, despite an increase in temperature, observed in specific regions of the Scandinavian countries and the Mediterranean islands (figure 5*d*).

Furthermore, we observed a clear decrease in the number of suitable months for *Ae. albopictus* in southern Spain, and also in southern Greece, attributed to higher summer temperatures (figure 5*a*, electronic supplementary material, figure S8). The negative impact of climate change in the suitability index, $R_{M}$, was more apparent in *Ae. albopictus* than in *Ae. aegypti* due to the lower thermal maximum of the former (figure 1*a*) particularly in Southern Europe and during the period 2061–2080.

## 4. Discussion

Mechanistic models grounded in thermo-biology insights have frequently been utilized to calculate the basic reproduction number ($R_{0}$) of vector-borne diseases [6,42]. Yet their use in evaluating the dynamics of vector populations themselves has been relatively limited [48], with statistical models typically being employed for this purpose. Understanding the distinction between reproductive numbers for vectors and diseases, as highlighted in previous literature [9], is crucial. Mosquito-borne disease cases are primarily mitigated by controlling mosquito populations. Therefore, calculating and visualizing $R_{M}$ independently from $R_{0}$ (which includes the disease component) can be valuable from a management perspective. The utility of both $R_{M}$ and $R_{0}$ in assessing mosquito-borne risk suggests that treating them as separate entities, with distinct names, is both necessary and justified.

Statistical suitability models infer the likelihood of observing a vector, relying on resource-intensive occurrence data [14,50,51]. Conversely, a suitability framework based on the basic reproductive number [8] offers a more precise delineation of temperature ranges and optima, directly depicting the potential for vector population growth under specific environmental conditions. The advantages of computing $R_{M}$ compared with other vector suitability models include its conceptual simplicity, rapid scalability and the potential for refinement over time, especially through the enhancement of thermal-response functions in laboratory settings and the application of downscaling methods to better assess local environmental conditions.

A strong limitation of the $R_{M}$ computation is the extrapolation of laboratory results (based on highly localized temperature data) to broader, averaged estimates of climatic variables over more extensive spatial areas. Other limitations of the $R_{M}$ computation become evident in its reliance on fixed-temperature laboratory experiments to estimate life cycle trait responses, which do not capture natural temperature fluctuations. Recent studies are addressing this issue by exploring the impacts of temperature fluctuations [11]. Additionally, considering other factors influencing vector life cycles, such as rainfall, relative humidity [19], human density and landscape features [18], may contribute to more realistic depictions of basic reproductive numbers and population dynamics. Building on Metelmann *et al.* [48], we used rainfall and human density as proxies for mosquito breeding site proliferation, impacting key life cycle parameters of *Aedes* [18,52], like the egg hatching rate and the carrying capacity. Since the $R_{M}$ describes the onset of the next generation from a small initial one, only the egg hatching rate plays a role whereas the carrying capacity vanishes from the $R_{M}$ computation. Of note, in our model, human density does not influence biting rates but only breeding site availability, which can fluctuate seasonally and geographically.

An important factor in the spread of *Aedes albopictus* to new regions is its ability to produce diapausing eggs at the end of the season, enabling survival through cold winters and fast recolonization in spring [53]. While some studies report improved model performance when including a diapause stage [54], we have opted to exclude this trait from our model. This decision is based on the fact that incorporating a diapause egg stage would not influence our $R_{M}$ results. The reason is that $R_{M}$ reflects the outcome for the next generation of mosquitoes in a specific area under given environmental conditions, following the arrival of a colonizing female. Consequently, the previous history of diapausing eggs cannot influence this computation (see electronic supplementary material, section 3, for more details). However, other calculations from similar ODE systems might yield different suitability indices where the diapause effect could be relevant, though this is not the case for $R_{M}$.

Our suitability maps show extensive favourable months for *Ae. albopictus* along the Mediterranean coast, aligning with recent findings of winter activity of this species [49] in Mediterranean regions. More generally, the current temperatures in Europe appear to be more favourable for *Ae. albopictus* compared with *Ae. aegypti*, with the latter exhibiting a higher temperature optimum than the former. This thermal suboptimal scenario may hinder the establishment of *Ae. aegypti* in Europe [55]. Comparing current decades (2020) with 2061–2080, most of Europe may see at least a one-month rise in suitability for both species, except parts of Scandinavia. Conversely, mountainous areas like the Alps or Pyrenees could experience a four- to seven-month increase. Northwestern countries like England and Ireland may also see a four-month rise of suitability for *Ae. albopictus*, and up to three months for *Ae. aegypti*. However, some coastal Mediterranean regions may face up to a six-month decrease in suitability due to temperatures surpassing the thermal limit for *Ae. albopictus* [56].

Overall, climate change may facilitate the establishment of *Ae. albopictus* in northern Europe and *Ae. aegypti* in southern Europe. While temperature rise generally favours these species, a substantial increase in summer temperatures could impact their suitability [57]. The temperature increase may result in unsuitable conditions during summer months, potentially disrupting the mosquito season and affecting mosquito dynamics by reducing their abundance or even leading to extinction, as they may struggle to thrive after such a drastic temperature surge. *Ae. aegypti* might be less sensitive to the impact of summer heat waves compared with *Ae. albopictus* due to its higher thermo-tolerance. Additionally, decreased rainfall in the future could further threaten both mosquitoes species by diminishing breeding sites. Finally, in areas suitable for both species, competitive interactions could influence their establishment rates and final distribution. For instance, *Ae. albopictus* larvae take longer to pupate when raised alongside *Ae. aegypti* [58]. While larval competition negatively affects the adult longevity of *Ae. aegypti*, it appears to have little impact on *Ae. albopictus* [59]. Conversely, some studies suggest that the presence of *Ae. aegypti* can reduce *Ae. albopictus* survival [60]. Thus, competitive interactions may shape the thermal responses of these species when they cohabit or attempt to colonize the same area.

When evaluating potential distributions of invasive species, it is also crucial to acknowledge their high behavioural plasticity [61]. For example, *Aedes* urban species have rapidly adapted to breed in artificial containers produced by humans. More specifically, *Ae. albopictus* has exhibited remarkable adaptability to new environmental conditions [62,63], including car-mediated dispersal by adults [64]. In the case of mosquitoes, rapid evolution [65,66] is a prominent factor, meaning that adaptive behaviours are rapidly fixed and spread over the populations. All in all, it is evident that future suitability and spatial distribution cannot be exclusively predicted by environmentally driven population dynamics, but must also consider the adaptive responses of mosquitoes within ever-expanding humanized landscapes.

While recognizing the limitations of basic vector indicators assessing growth suitability, we suggest $R_{M}$ maps can prove a practical strategy for vector surveillance and proactive measures in regions vulnerable to urban *Aedes* mosquito proliferation.

## Data Availability

The data supporting the results that are not open and accessible through dedicated web pages and from other literature cited in the main manuscript are the following datasets, all uploaded to the Dryad repository [34]: (i) *Ae. albopictus* presence–absence data at the municipality level for Spain, (ii) presence–absence data for *Ae. albopictus* at NUTS3 level for Europe, and (iii) *Ae. albopictus* trap count data from the period 2018–2022 in Spain uploaded to the same repository. The R code used for processing the data and computing the suitability maps is available in the Zenodo repository [67]. A preprint of this manuscript is available [68]. Supplementary material is available online [69].
